# Comparison of cardiac index measurements in intensive care patients using continuous wave vs. pulsed wave echo-Doppler compared to pulse contour cardiac output

**DOI:** 10.1186/s40635-023-00499-2

**Published:** 2023-04-28

**Authors:** Prashant Parulekar, James Powys-Lybbe, Paul Bassett, Seb Roques, Mark Snazelle, Gemma Millen, Tim Harris

**Affiliations:** 1 Intensive Care and Acute Medicine, East Kent Hospitals University NHS Foundation Trust, William Harvey Hospital, London, UK; 2 Royal Brompton and Harefield Trust, London, UK; 3 StatConsultancy Ltd, Amersham, UK; 4 East Kent Hospitals University NHS Foundation Trust, William Harvey, London, UK

**Keywords:** Echocardiography, Intensive care, Doppler, Ultrasound

## Abstract

**Purpose:**

Cardiac index (CI) assessments are commonly used in critical care to define shock aetiology and guide resuscitation. Echocardiographic assessment is non-invasive and has high levels of agreement with thermodilution assessment of CI. CI assessment is derived from the velocity time integral (VTI) assessed using pulsed wave (PW) doppler at the level of the left ventricular outflow tract divided by body mass index. Continuous wave (CW) doppler through the aortic valve offers an alternative means to assess VTI and may offer better assessment at high velocities.

**Methods:**

We performed a single centre, prospective, observational study in a 15-bed intensive care unit in a busy district general hospital. Patients had simultaneous measurements of cardiac index by Pulse Contour Cardiac Output (PiCCO) (thermodilution), transthoracic echocardiographic PW-VTI and CW-VTI. Mean differences were measured with Bland–Altman limits of agreement and percentage error (PE) calculations.

**Results:**

Data were collected on 52 patients. 71% were supported with noradrenaline with or without additional inotropic or vasopressor agents. Mean CIs were: CW-VTI 2.7 L/min/m^2^ (range 0.78–5.11, SD 0.92). PW-VTI 2.33 L/min/m^2^ (range 0.77–5.40, SD 0.90) and PiCCO 2.86 L/min/m^2^ (range 1.50–5.56, SD 0.93). CW-VTI and PiCCO mean difference was − 0.16 L/min/m^2^ PE 43.5%. PW-VTI and PiCCO had a mean difference of − 0.54 L/min/m^2^ PE 38.6%. CW-VTI and PW-VTI had a mean difference of 0.38 L/min/m^2^ PE 46.0%.

**Conclusions:**

CI derived from both CW-VTI and PW-VTI methods underestimate CI compared to PiCCO, with the CW-VTI method having closer values overall to PiCCO. CW-VTI may offer a more accurate assessment of CI. If using Critchley’s PE cutoff of 30%, none of the doppler methods may accurately reflect the actual cardiac index.

## Introduction

Cardiac index (CI) assessments are commonly used in critical care to define shock aetiology and guide resuscitation. The pulmonary artery catheter (PAC) has been in use for over 50 years and remains the most widely accepted reference standard [1–4]. The PAC has been largely replaced in intensive care units (ICU) by less invasive methods, such as transpulmonary thermodilution, Pulse Contour Cardiac Output (PiCCO), oesophageal doppler and transthoracic echocardiography (TTE) [5–7].

TTE is increasingly used in critical care medicine, offering a comprehensive evaluation of cardiac function in addition to non-invasive CI and stroke volume index (SVI). In 1984, Lewis described the PW technique to measure left ventricular outflow tract (LVOT) velocity time integral (VTI) and calculate stroke volume (SV) [8]. Several other TTE-based approaches have been described to assess cardiac output (CO) with this method correlating with PAC most closely [9, 10]. The VTI may also be assessed using continuous wave (CW) Doppler, which assesses blood volumes passing through the aortic valve. CW technology deals better than pulsed wave (PW) with higher flow velocities, representing small proportions of the actual SV that may be excluded in PW calculation. Unlike PW, correlation of gate position for VTI measurement does not have to be considered but VTI may include erroneous velocities at any point along the ultrasound beam, such as opening and closing valve artefacts or LVOT obstruction. Evangelista [11] compared the reliability of CW and PW (using mean velocities) derived VTI for measuring CO against the PAC as reference standard. PW underestimated CI as compared to PAC derived values (− 28% ± 13%, *P* < 0.01), while CW mean values closely matched those obtained using PAC. CW derived values had poorer inter and intra observer variability.

The accuracy of PW-VTI as compared to PAC thermodilution has been well-studied. A 2015 study comparing PW-VTI to PAC using both thermodilution and Fick, reported minimal bias (0.1 L/min) but with large limits of agreement (LOA) between the latter [12]. The PW-VTI underestimated the CO by 0.3 l/min. Other studies have shown excellent correlation between PW-VTI and PAC thermodilution [13–15] PiCCO uses a combination of trans-cardiopulmonary thermodilution and pulse contour wave analysis to calculate SVI and CI. Previous studies have shown reasonable levels of accuracy and precision as compared to PAC [6, 16–18] and in one recent multicentre study PiCCO and PAC thermodilution were used as identical reference standards [19]. A recent meta-analysis reported both echocardiographic and PiCCO derived CO to have superior precision when compared to PAC as a reference standard as opposed to other minimally or non-invasive methods [20]. The PAPIKAS investigators reported PiCCO to have correlated well with PAC in patients with cardiac shock.

In this study, we assess the accuracy in measuring CI using CW-VTI as compared to PW-VTI against the a priori defined reference standard of PiCCO. We hypothesised that as CW assesses the VTI ejected through the AV, and offers a high-quality tracing over higher velocities, it would offer a more accurate assessment of CI.

## Methods

We performed a single centre, prospective, observational study in a 15-bed ICU of a district general hospital. The study was approved and consent to participate not deemed necessary, as all data were collected as part of routine care with no deviations from usual practice nor patient identifiable data obtained, by the Trust Research and Innovation (R&I) Department (Ref 2021/GAP/03).

We collected data on patients greater than 18 years with a PiCCO line in situ admitted 08/02/2019–27/01/2020 as part of routine clinical care (convenience series). Patients were excluded if they were known or identified to have any grade of aortic stenosis or aortic regurgitation (defined by British Society of Echocardiography (BSE) criteria [21]).

For calculation of the CI the heart rate at the time of performing the TTE was used for all assessments. Any differences in CI were consequently due to measurements of SV only. No change in management (such as additional fluid therapy, analgesia, inotropic medication doses) occurred between performing the PiCCO study and TTE studies. All TTEs were performed by one of three operators, each an intensive care medicine consultant with BSE level 2 training or Diploma of Echocardiography (Paris).

The PiCCO was calibrated by the nursing staff using thermodilution technique. Three calibrations were recorded (which is standard care) and the average readings noted. The researcher was blinded to the PiCCO results and performed the TTE directly after the PiCCO study.

CI subgroups were categorised using standard clinical guidelines of low CI (less than 2.5 L/min/m^2^) and high CI (greater than 4.0 L/min/m^2^). Timing subgroups were determined arbitrarily as less than 15 min and greater than 15 min between PiCCO reading and TTE measurements.

Stata (version 15.1) software was used for statistical analysis.

### Imaging

A Sonosite X-Porte ultrasound machine and a 2–5 MHz phased array transducer were used for all the studies. The key measurements obtained were the LVOT diameter and VTI using PW and CW. All measurements were obtained according to BSE criteria [21]. To obtain the LVOT diameter, a zoomed PLAX view of the LVOT was frozen with the diameter measured with the AV leaflets open. The PW-VTI was manually traced via a focused frozen apical 5-chamber view with the PW Doppler gate placed 3 mm to the LVOT. The CW-VTI was manually traced using a focused frozen apical 5-chamber view with the CW Doppler through the AV. The PW-VTI was measured first, followed by the CW-VTI [10, 11, 22, 23].

The highest quality images were selected by the researcher. An average of three images were obtained per patient in sinus rhythm, five if atrial fibrillation [21].

CI was calculated for each patient using the formula:

$${\text{CI }} = {\text{ CO}}/{\text{BSA}}$$

$${\text{CO }} = {\text{ SV }} \times {\text{ Heart rate}}$$

$${\text{SV }} = \, \left[ {\left( {{3}.{1416}} \right) \, \times \, \left( {{\text{LVOTd}}/{2}} \right)^{{2}} } \right] \, \times {\text{ VTI }}\left( {\text{CW or PW}} \right)$$

BSA = Patient’s body surface area

### Data collection

Demographic and clinical data including Acute Physiology and Chronic Health Evaluation II score (APACHE II), BSA (using the DuBois formula), diagnosis on ICU admission, use of mechanical ventilation and mode of ventilation including positive end expiratory pressure (PEEP) and fraction of inspired oxygen (FiO2), heart rate and rhythm were recorded directly into a Microsoft Excel database. All data were securely stored along Trust and NHS criteria.

### Statistical analysis

Critchley recommend that studies comparing CO monitors report mean CO, bias, LOA and percentage error (PE) [24]. The author suggested that to be clinically acceptable the monitor being assessed should report LOA of ± 30%: an acceptable range by which two methods of measurement, each with a notional error of approximately 10–20%, can be considered interchangeable. Bland–Altman (B–A) analysis was used to assess LOA. Bias was defined as the mean difference between CI measurements by each set of paired devices (PiCCO: CW-VTI, PiCCO: PW-VTI and CW-VTI: PW-VTI) and was plotted against the mean of the two values (bias ± 1.96 standard deviations of differences between methods). A statistical comparison of the agreement with the reference standard between CW-VTI CI and PW-VTI CI was made. The absolute difference with the reference standard (i.e., magnitude of the difference regardless of the direction) was compared between groups using the paired *t* test.

The use of PE compensates for the widely differing ranges of CI that may be identified in any study comparing devices. A difference of 1 L/min between two devices represents a more significant error for a low as opposed to a high CI. The PE was calculated as limits of agreement divided by the mean CO, as below:

$${\text{Percentage error}} = \frac{{\left( {1.96\, \times \left( {\text{Standard Deviation of bias between two methods}} \right)} \right)}}{{0.5 \times \left( {\text{Mean non - invasive Cardiac Output + mean PiCCO Cardiac Output}} \right)}}$$ [25]

## Results

Data were obtained on 55 patients (08/02/2019–27/01/2020), with three patients omitted due to aortic stenosis (AS) detected on TTE and no patients having LVOT obstruction. Two patients required assistance with an intra-aortic balloon pump; however, there were no patients on cardiac support devices at the time the results were taken. Therefore, 52 patients were included with simultaneous measurements of CI by PiCCO, PW-VTI and CW-VTI, all with full data sets. Diagnostic images were obtained on all patients admitted during the study period. Patient characteristics are shown in Table 1. 63% were admitted to critical care post cardiac arrest, 80% were mechanically ventilated and the median APACHE II was 16.

**Table 1:** Characterisation of patients

Category	Patients (*n* = 52)
Age (years), median (p25–p75)	61 (54–72)
APACHE II, median (p25–p75)	16 (13–20)
Body Surface Area (m^2^), median (p25–p75)	1.99 (1.91–2.06)
Reason for ICU admission, number (%)	
Out of hospital cardiac arrest Acute respiratory failure Cardiogenic shock Other	33 (63%) 5 (10%) 2 (4%) 12 (23%)
Respiratory support, number (%)	
Oxygen via mask Non-invasive ventilation Mechanical ventilation Positive end expiratory pressure (cmH_2_O) Driving pressure (mmHg)	3 (6%) 7 (14%) 42 (80%) 7 (5–8) 11 (8–14)
Past medical history, number (%)	
Diabetes type 2 Hypertension Smoker Ethanol Transplant (stem cell) Stroke Angina Coronary artery bypass surgery Coronary artery disease Pulmonary embolism Chronic kidney disease Chronic obstructive pulmonary disease Asthma	4 (8%) 17 (33%) 14 (27%) 7 (13% 1 (2%) 1 (2%) 1 (2%) 1 (2%) 2 (5%) 4 (8%) 1 (2%) 8 (15%) 2 (5%)
Interventions, number (%)	
Invasive ventilation Targeted temperature management completed Hypothermia Intra-aortic balloon pump	42 (81%) 34 (65%) 0 2 (5%)
Vasopressors/inotropes, number (%)	
Noradrenaline Argipressin Dobutamine Levosimendan	37 (71%) 7 (13%) 12 (23%) 11 (21%)
Arrythmias/malignant Arrythmias, number (%)	
Atrial fibrillation Atrial flutter Ventricular fibrillation Ventricular tachycardia	7 (13%) 1 (2%) 1 (2%) 8 (15%)

*APACHE II* acute physiology and chronic health evaluation II score

The CI values assessed using PW-VTI, CW-VTI and PiCCO are reported in Table 2. Data are presented as means with SD. Normal data distribution was confirmed by visual assessment. The summary statistics suggest similar mean CI values between the CW-VTI and PiCCO method, with lower values for the PW-VTI method. The spread of values, as indicated by the SD, was similar for all three measurements. The absolute difference between each TTE-based method and PiCCO is also shown in Table 2. The results show a non-significant difference between the two TTE assessments of CI and PiCCO.

**Table 2:** Summary of cardiac index results including mean, standard deviation, range and absolute difference with PiCCO mean

Measurement	No.	Mean (L/min/m^2^)	Standard deviation (L/min/m^2^)	Range (L/min/m^2^)
CW-VTI CI	52	2.70	0.92	0.78, 5.11
PW-VTI CI	52	2.33	0.90	0.77, 5.40
PiCCO CI	52	2.86	0.93	1.50, 5.56

Measurement	Absolute difference with PiCCO Mean ± Standard deviation (L/min/m^2^)	*P* value
CW-VTI CI	0.47 ± 0.43	0.07
PW-VTI CI	0.61 ± 0.41	

*N* number of patients; *CI* cardiac index; *CW-VTI* continuous-wave velocity time integral; *CW-VTI* pulsed-wave velocity time integral; *PiCCO* pulse contour cardiac output

**Fig. 1:**
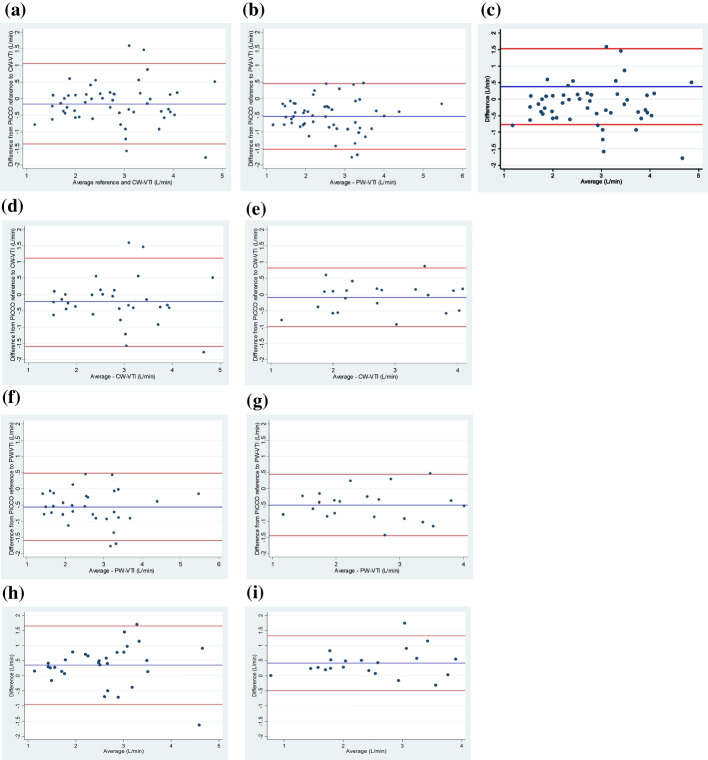
Panel figure of Bland–Altman plots. **A** Bland–Altman plot for CW-VTI measurement relative to PiCCO reference; **B** Bland–Altman plot for PW-VTI measurement relative to PiCCO reference; **C** Bland–Altman plot for CW-VTI measurement relative to PW-VTI measurement. (Continuous-wave Velocity Time integral = CW-VTI; Pulsed-wave Velocity Time integral = CW-VTI; Pulse Contour Cardiac Output = PiCCO); **D** Bland-Altman plot for CW-VTI measurement relative to PiCCO reference–Measurements less than 15 min only; **E** Bland-Altman plot for LVOT measurement relative to PiCCO reference–Measurements less than 15 min only; **F** Bland-Altman plot for CW-VTI measurement relative to PW-VTI measurement–Measurements less than 15 min only; **G** Bland-Altman plot for CW-VTI measurement relative to PiCCO reference–Measurements greater than 15 min only; **H** Bland-Altman plot for PW-VTI measurement relative to PiCCO reference–Measurements greater than 15 min only; **I** Bland-Altman plot for CW-VTI measurement relative to PW-VTI measurement–Measurements greater than 15 min only

B–A LOA for each of the TTE methods and PiCCO, and between PW-VTI and CW-VTI are summarised in Table 3 and displayed in Fig. 1. The results show a smaller difference between CW-VTI and PiCCO assessment of CI than with PW-VTI and PiCCO: − 0.16 L/m^2^/min vs. − 0.54 L/m^2^/min. The B–A LOA for the AV CW-VTI and PiCCO measurements were approximately ± 1.2 L/m^2^/min. The width of the LOA for the PW-VTI and PiCCO measurements were slightly narrower than those for CW-VTI. PE calculations shows slightly reduced error with PW-VTI as compared to CW-VTI.

**Table 3:** Primary analysis and subgroup analysis by time, including mean differences between measurements, the standard deviation of the differences, the 95% Bland–Altman limits of agreement and percentage error

Measurements	No.	Mean difference (L/min/m^2^)	Standard deviation difference (L/min/m^2^)	95% Bland–Altman limits (L/min/m^2^)	Percentage error (%)
CW-VTI CI and PiCCO CI^a^	52	− 0.16	0.62	(− 1.37, 1.05)	43.5
PW-VTI CI and PiCCO CI^a^	52	− 0.54	0.51	(− 1.53, 0.46)	38.6
CW-VTI CI and PW-VTI CI^b^	52	0.38	0.59	(− 0.77, 1.52)	46.0
Less than 15 minutes					
CW-VTI CI and PiCCO CI^a^	31	− 0.21	0.71	(− 1.60, 1.18)	49.0
PW-VTI CI and PiCCO CI^a^	31	− 0.56	0.53	(− 1.60, 0.48)	39.1
CW-VTI CI and PW-VTI CI^b^	31	0.35	0.66	(− 0.95, 1.65)	50.7
Greater than 15 min					
CW-VTI CI and PiCCO CI^a^	21	− 0.08	0.46	(− 0.99, 0.82)	33.2
PW-VTI CI and PiCCO CI^a^	21	− 0.50	0.48	(− 1.44, 0.44)	37.6
CW-VTI CI and PW-VTI CI^b^	21	0.42	0.46	(− 0.49, 1.32)	36.6

*N* Number of patients; *CI* Cardiac Index; *CW-VTI* continuous-wave velocity time integral; *CW-VTI* pulsed-wave velocity time integral; *PiCCO* pulse contour cardiac output

^a^Differences calculated as values for CW-VTI or PW-VTI minus value for PiCCO

^b^Differences calculated as values for CW-VTI minus value for PW-VTI

The agreement between the TTE methods and each to the reference standard PiCCO were examined in timing subgroups as either less than or greater than 15 min between PiCCO and the TTE (Table 3 and Fig. 1). The figures presented are mean differences between measurements, the SD of the differences and the 95% B–A LOA. The results show no significant difference between the time subgroups.

A sensitivity analysis was performed using B–A LOA for cardiac rhythm and low or high CI (Table 4). The results for the sinus rhythm patients are almost identical results to the results for the whole cohort. Omitting the two AF patients made little difference to the results.

**Table 4:** Subgroup analysis for rhythm and cardiac index including mean differences between measurements, the standard deviation of the differences and the 95% Bland–Altman limits of agreement

Measurements	No.	Mean difference (L/min/m^2^)	Standard deviation difference (L/min/m^2^)	95% Bland–Altman limits (L/min/m^2^)
Sinus rhythm				
CW-VTI CI and PiCCO CI^a^	50	− 0.17	0.63	(− 1.40, 1.07)
PW-VTI CI and PiCCO CI^a^	50	− 0.53	0.51	(− 1.54, 0.47)
High CI (> 4 L/min)				
CW-VTI CI and PiCCO CI^a^	7	− 0.57	0.69	(− 1.93, 0.78)
PW-VTI CI and PiCCO CI^a^	7	− 0.95	0.63	(− 2.19, 0.29)
Low CI (< 2.5 L/min)				
CW-VTI CI and PiCCO CI^a^	22	− 0.04	0.52	(− 1.06, 0.98)
PW-VTI CI and PiCCO CI^a^	22	− 0.39	0.36	(− 1.10, 0.44)

*N* Number of patients; *CI* Cardiac Index; *CW-VTI* Continuous-wave Velocity Time integral; *CW-VTI* Pulsed-wave Velocity Time integral; *PiCCO* Pulse Contour Cardiac Output

^a^Differences calculated as values for CW-VTI minus value for PiCCO

^b^Differences calculated as values for CW-VTI minus value for PW-VTI

## Discussion

In this single centre, observational study of 52 patients, diagnostic images and complete data set were obtained on all participants. The mean CI values for CW-VTI and PiCCO were similar (2.70 L/min/m^2^ and 2.86 L/min/m^2,^ respectively), with lower mean values identified for PW-VTI (2.33 L/min/m^2^). The mean difference (bias) between CW-VTI and PiCCO was − 0.16 L/min/m^2^ and between PW-VTI and PiCCO − 0.54 L/min/m^2^. This continued over a wide range of CIs suggesting that CW-VTI may offer a more accurate estimate than PW-VTI, using PiCCO as a reference standard, while PW-VTI underestimates CI as compared to both PiCCO and CW-VTI with a mean difference (bias) of − 0.38 L/min/m^2^.

These results are similar to Evangelista [11], where CW mean values closely matched those obtained using PAC and PW underestimated CO as compared to PAC derived values (− 28% ± 13%, *P* < 0.01).

There is no gold standard for clinicians to assess CI and the PAC remains the most widely used reference standard. PAC is rarely used so in this paper the PiCCO (thermodilution) was designated as the reference standard. PiCCO as a reference device is open to question with a recent meta-analysis suggesting that the PW-VTI offered a better agreement: tolerability index and summary percentage errors to PAC than PiCCO (using thermodilution) to PAC [20]. However, the paper did report a smaller spread of values for PiCCO vs. PAC and using a reference device assists in the comparison between CI as assessed by CW-VTI and PW-VTI. The PAC (thermodilution) does not allow continuous CI monitoring, is influenced by ventilation, core temperature and valvular disease, and, traditionally, requires manual input. Previous work reported a PE of 25% for PW-VTI compared with PAC [10], and a bias of − 0.2 L/min (CO not CI) with LOA − 1.2, 1.8 L/min, like this paper.

Most clinicians would not regard the CW-VTI difference of 0.16 L/min/m^2^ as clinically significant but may the 0.54 L/min/m^2^ mean difference between PiCCO and PW-VTI, as this value may be above the 10–15% increase in CI that defines a fluid responder to a 500 ml fluid challenge. One possible explanation for CW-VTI derived CI values being closer to PiCCO derived values is a more accurate tracing of red blood cells, the VTI “envelope”, using the CW doppler as opposed to using the gated PW doppler.

We showed a difference approaching statistical significance between the two methods in terms of their absolute mean difference from the PiCCO derived CI, *p* = 0.07. The absolute mean difference comparing CW-VTI CI to PiCCO CI was 0.47 ± 0.43 L/min/m^2^, as opposed to the absolute mean difference of 0.61 ± 0.41 L/min/m^2^ comparing PW-VTI CI to PiCCO CI. With no power calculation available the significance of this finding is open to debate. On average, the results again indicate CI values via the CW-VTI method to be closer to the PiCCO method CIs than those via the PW-VTI method.

There were two cases which were outliers, where the PW-VTI CI values were considerably lower than the PiCCO CI, with differences being 1.80 and 1.70 L/min/m^2^. These are significant differences, and these outlier results may have impacted the accuracy of the PW derived method in overall differences from the PiCCO method.

In this study the spread of the values (standard deviations) were similar for all three methods, suggesting similar precision. The B–A limits for the CW-VTI and PiCCO measurements were approximately ± 1.2 L/m^2^/min from the mean value. Thus, while the mean values for CI obtained suggest CW-VTI and PiCCO may be used interchangeably the wide LOA may not support this. 95% of the values obtained by CW-VTI will be within 1.2 L/m^2^/min of the mean value obtained by PiCCO, so potentially seeing the two methods classifying CI differently, one as normal and the other method as abnormal.

The 95% B–A LOA were slightly smaller for PW-VTI and PiCCO (± 1.0 L/m^2^/min) than CW-VTI and PiCCO, indicating slightly increased precision of the PW method. However, due to the larger mean difference between PW-VTI and PiCCO, the B–A LOA are skewed towards lower values. The bottom range LOA suggests that the CI could be up to 1.5 L/m^2^/min lower for the PW-VTI method than the PiCCO derived assessment, risking misclassification of shock syndromes and instituting inappropriate care.

Previous authors have used a range of methods to assess and compare CI measuring devices. Accuracy (bias, the difference or systematic error between assessed techniques) and precision (the scatter or random error between techniques, LOA) are best expressed using B–A graphs [26]. Critchley suggested acceptable LOA of 10–20% (errors consequent upon respiratory cycle variation in CI and lack of precision of device, with three to five averaged readings to minimise these), based on the assumed accuracy of PAC, or, 30%, based on the notional error of the two techniques under review [24]. The PE adjusts the LOA for CI and given the high proportion of low CI patients in this study enables wider comparison. It provides assessment of the changes in CI not linked to true changes in CI but to error within the two systems under comparison.

In the paper published today the PE were: 43.5% (CW-VTI–PiCCO), 38.6% (PW-VTI–PiCCO) and 46.0% (PW-VTI–CW-VTI). These are all higher than the recommended maximum value of 30% suggested by Critchley in 1999. However, Peyton and Chong performed a meta-analysis in 2010 that concluded a limit of 30% was somewhat arbitrary and does not reflect in vivo PE in a range of invasive and non-invasive methods of CI monitoring devices. Their recommendation is that a PE of 45% may be more suitable threshold, which was met by both methods in this study [25].

## Limitations

The TTE was performed as soon as possible to the PiCCO calibration with thermodilution; however, this was not always feasible immediately. Ideally, to minimise confounding factors, the TTE and calibrated PiCCO should be performed at the same time. However, no additional therapies were performed (such as a fluid bolus or additional inotropy) in between the calibrated PiCCO and TTE. The sensitivity analysis performed for TTE scans taken less than or greater than 15 min post PiCCO studies suggested no significant difference in the two methods compared to PiCCO.

For SV calculation for this study, we used the LVOT diameter for calculation of both the SV derived using PW-VTI and CW-VTI measurements. The reason for this is that AV area calculation is derived using the LVOT diameter and so for consistency this measurement was used for all SV calculations. CI was used rather than SV as CI is more important for oxygen delivery rather than SV, also the CI was automatically calculated with PiCCO making comparison with the TTE findings more practical.

A significant proportion of patients in the study had low CIs (22/52 patients having a CI < 2.5 L/min) and our findings may not apply to non-ventilated patients and those with higher CI. As a high proportion of patients were admitted after cardiac arrest this may limit generalisability of the results.

We cannot comment on the ability to track fluid responsiveness or to track trends in CI with treatment and/or time, as we did not include serial measurements in this study. Consistency of response is arguably more important to clinicians than absolute values, and subject to the precision of the device—which we did not look at here either.

This paper would also have been improved by the evaluation of inter and intra observer agreement for doppler measurements and taking repeated measurements using PiCCO thermodilution to assess the precision of the device.

## Conclusions

In this study CI derived from both CW-VTI and PW-VTI methods underestimate CI compared to PiCCO, with the CW-VTI assessment having closer values to the PiCCO. CW-VTI may offer a more accurate assessment of CI than the current practice of using PW-VTI to assess CI via TTE. However, this study is single centre with no assessment of inter- or intra-observer reliability. If using Critchley’s PE cutoff of 30%, each doppler method may not reliably reflect the true cardiac index. More work is required to clarify the most accurate, precise, and reproducible method to assess CI using TTE.

## Take home message

Cardiac index calculated by transthoracic echocardiography is closer to the PiCCO reference standard when the velocity time integral is measured by continuous wave doppler across the aortic valve than it is by pulse wave measurement at the left ventricular outflow tract.

## 140 character tweet

CI calculated by TTE is closer to PiCCO reference when the VTI is measured by continuous wave compared to pulse wave doppler.

## Data Availability

Original anonymised data may be available on request.

## Abbreviations

CW-VTI: Velocity–time-integral by continuous wave technique
PW-VTI: Velocity–time-integral by pulsed wave technique
VTI: Velocity time integral
CI: Cardiac index
PW: Pulsed wave
CW: Continuous wave
PiCCO: Pulse Contour Cardiac Output
PE: Percentage error
PAC: Pulmonary artery catheter
ICU: Intensive care units
TTE: Transthoracic echocardiography
SVI: Stroke volume index
LVOT: Left ventricular outflow tract
SV: Stroke volume
CO: Cardiac output
LOA: Limits of agreement
BSE: British Society of Echocardiography
BSA: Body surface area
PEEP: Positive end expiratory pressure
FiO2: Fraction of inspired oxygen
B–A: Bland–Altman
AS: Aortic stenosis
SD: Standard deviations
